# Variable Holocene deformation above a shallow subduction zone extremely close to the trench

**DOI:** 10.1038/ncomms8607

**Published:** 2015-06-30

**Authors:** Kaustubh Thirumalai, Frederick W. Taylor, Chuan-Chou Shen, Luc L. Lavier, Cliff Frohlich, Laura M. Wallace, Chung-Che Wu, Hailong Sun, Alison K. Papabatu

**Affiliations:** 1 Institute for Geophysics, Jackson School of Geosciences, University of Texas at Austin, J. J. Pickle Research Campus, Building 196, 10100 Burnet Road (R2200), Austin, Texas 78758, USA; 2 Department of Geological Sciences, Jackson School of Geosciences, University of Texas at Austin, 1 University Station C9000, Austin, Texas 78712, USA; 3 Department of Geosciences, High-Precision Mass Spectrometry and Environment Change Laboratory (HISPEC), National Taiwan University, Taipei, Taiwan 10617; 4 The State Key Laboratory of Environmental Geochemistry, Institute of Geochemistry, Chinese Academy of Sciences, Guiyang 550002, China; 5 Department of Mines, Energy, and Water Resources, PO Box G37, Honiara, Solomon Islands

## Abstract

Histories of vertical crustal motions at convergent margins offer fundamental insights into the relationship between interplate slip and permanent deformation. Moreover, past abrupt motions are proxies for potential tsunamigenic earthquakes and benefit hazard assessment. Well-dated records are required to understand the relationship between past earthquakes and Holocene vertical deformation. Here we measure elevations and ^230^Th ages of *in situ* corals raised above the sea level in the western Solomon Islands to build an uplift event history overlying the seismogenic zone, extremely close to the trench (4–40 km). We find marked spatiotemporal heterogeneity in uplift from mid-Holocene to present: some areas accrue more permanent uplift than others. Thus, uplift imposed during the 1 April 2007 *M*_w_ 8.1 event may be retained in some locations but removed in others before the next megathrust rupture. This variability suggests significant changes in strain accumulation and the interplate thrust process from one event to the next.

## Abstract (web summary)

Information regarding tectonic motion from before instrumental records can be found from palaeoshorelines and the reconstruction of sea level from observations. Here, the authors study corals uplifted by past earthquakes near the Solomon Islands and assess the Holocene deformation that took place there.

## Graphical abstract

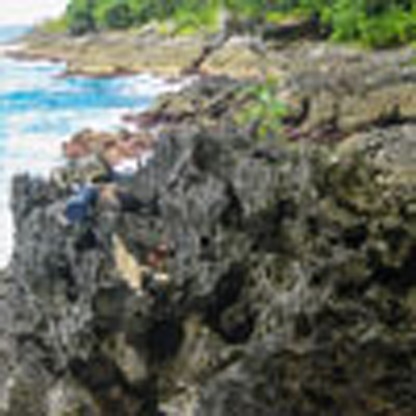

On 1 April 2007, a large *M*_w_ 8.1 earthquake in the Western Province of the Solomon Islands ruptured across the Australian-Woodlark-Pacific triple junction^1^,^2^,^3^,^4^, produced ≥2.5 m of uplift^1^, generated a 12-m tsunami^5^ and killed over 50 people^6^. Subduction at this margin is complex because of the very young (0.5–3 Ma) and rugged sea-floor topography^7^ and segmentation of the downgoing plate^8^. The Australian plate, conveying the Simbo ridge and transform system, and the Coleman and Kana Keoki volcanic seamounts (Supplementary Fig. 1), converges with the Pacific plate at ∼97 mm per year (yr) towards N 70°E (ref. 9; Fig. 1). Northwest of the Simbo transform, the Woodlark Plate converges at a higher rate of ∼108 mm per yr nearly orthogonal to the arc trend^1^. The 2007 event began near the Australian plate-Solomons forearc megathrust surface, crossed the Simbo transform fault and then ruptured along the Woodlark-western forearc boundary. Seismological analysis of the 2007 rupture, local seismic reflection and ocean bottom seismometer surveys indicate a relatively shallow interplate thrust zone dipping northeastward from the trench floor to a depth of 20 km beneath the arc^2^,^4^,^10^. Uplifted coral ages indicate that the entire forearc is undergoing accelerated net uplift, initiated after 50 ka (ref. 11). However, the link between this long-term forearc deformation and interplate slip, whether coseismic or otherwise, in the western Solomons is unclear.

Coral paleogeodesy provides the key insights into the mechanisms through which the overriding plate accommodates interplate convergent slip: coseismic or otherwise^12^,^13^,^14^,^15^ (Fig. 2). Corals provide measurements of both the timing and amounts of previous abrupt vertical motions that sum over time to tens or hundreds of metres of net uplift that dominate the morphotectonic evolution of forearcs^16^,^17^,^18^,^19^. The application of coral paleogeodesy to investigate the vertical tectonic history of the western Solomons is particularly suitable because of the following: (1) rapid plate convergence and tectonic uplift rates, (2) the opportunity to take measurements on islands positioned directly above the shallow megathrust rupture zone, an essential location for understanding interplate seismogenic and deformation processes^20^, (3) the well-measured coseismically uplifted 2007 shoreline^1^ and the clearly delineated, elevated mid-Holocene shoreline^11^ and (4) the fact that before the 2007 event the region was characterized as having aseismic convergence and weak coupling. No events larger than *M*_w_ 7.2 were instrumentally recorded between 156°E and 158°E from 1900 to 2007 (refs 8, 21, 22). Thus, the 2007 event demonstrates that great earthquakes can occur in the western Solomons and that paleogeodetic uplifts provide a precisely datable proxy for past earthquake chronology, geography and recurrence intervals that are vital for understanding the role of megathrust earthquake cycles in arc tectonics and for assessing risk^12^,^13^,^14^,^15^,^23^.

In this work, using the elevation and ages of uneroded, *in situ* corals, we reconstruct late Holocene vertical deformation at sites on land located closer to the trench (within 4–40 km from the trench) than in any previous study. U-Th chemistry^24^,^25^ of the outer coral layers was used to determine the absolute ages (reported in years before 2012) of abrupt uplift events that killed these corals (Fig. 2). By comparing the abrupt uplift profile reconstructed from *in situ* coral levels (see Methods) with longer-term net uplift^11^, we find evidence for pronounced spatiotemporal variability in the evolution of Holocene deformation at the western Solomon Islands. The reconstruction also indicates that prehistoric earthquake events imposed uplifts larger than or comparable to the 2007 event.

## Results

### Reconstructed uplift profile

**Figure 1:**
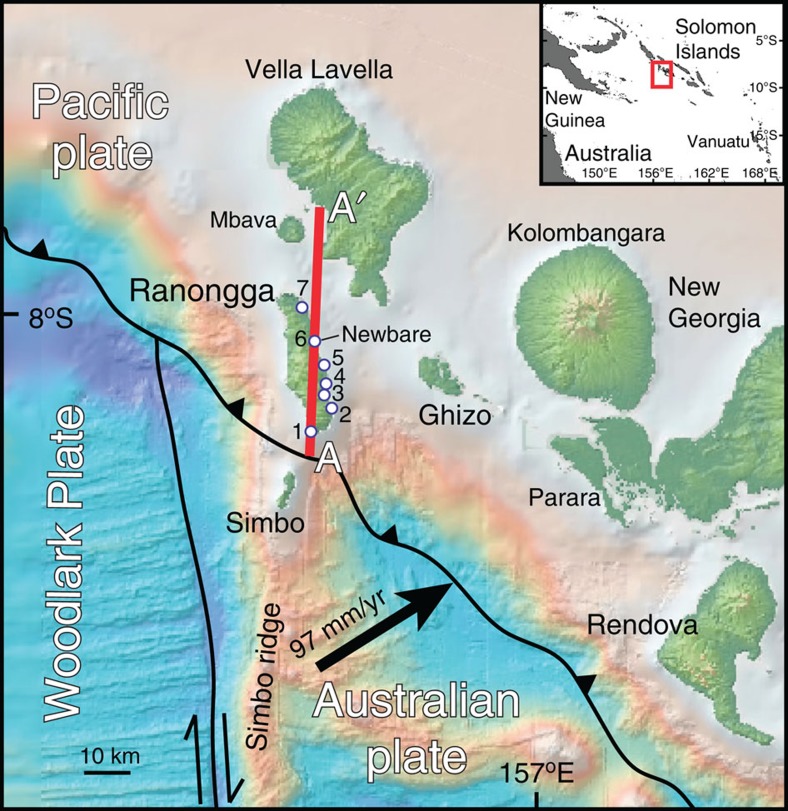
Tectonic setting of the western Solomon Islands' subduction zone. White circles indicate *in situ* coral sampling sites: 1—Lale, 2—Konggu, 3—Perava Pt., 4—Ena, 5—Ndae, 6—Newbare, 7—Kolomali. Bathymetric base map from the Global Multi-Resolution Topography synthesis^43^.

Along line AA′ (Fig. 1), extending through Ranongga and Vella Lavella, we plot the elevations of uplifted corals on three timescales (Fig. 3a): (1) the amount of 2007 coseismic uplift^1^ (red stars; Supplementary Table 3), (2) the total emergence since the mid-Holocene^11^ (blue stars; Supplementary Table 4) and (3) *in situ*, dated coral uplift levels described in the Methods section (blue circles decreasing in size with age; this study—Supplementary Tables 1 and 2). Previous ^14^C dates of the uppermost Holocene reef flat at ∼6 ka (ref. 11; ages reported in years before 1950) and our dates of 6–7 ka demonstrate its mid-Holocene age. Depending on the age obtained for a particular coral sample, we corrected for paleosea level changes in the Western Solomons using the well-documented Western Pacific Mid-Holocene highstand^26^,^27^,^28^,^29^,^30^ where regional sea level reached 1–2 m higher than at present compared with ∼5–6.5 ka. The exact timing and magnitude of this highstand vary widely from study-to-study, but for our analysis we chose +2 m at 6 ka. Whether the actual amount was +0.5 or +1 m and whether it occurred 1,000 yrs earlier or 1,000 yrs later than 6 ka is negligible to our interpretation because of the relatively large amounts of uplift imposed on the corals since the Holocene (Supplementary Table 2).

Multiple uplifted levels less than 6,000 years old were identified at most sample sites. Corals from Konggu (labelled 2 in Figs 1 and 3) and Kolomali (labelled 7 in Figs 1 and 3), which are 19 km apart and 2.06 and 1.63 m above the 2007 level, respectively, date the penultimate (pre-2007) abrupt uplift at ∼600 years ago. We have not found ∼600-yr-old corals at other locations; however, if any were uplifted less than ∼1 m above living corals they may have been eroded away. At Newbare (labelled 6 in Fig. 1), the site containing the longest sequence of abrupt events, the first corals above the 2007 level are 3.4 m higher and 760 yrs old rather than ∼600 yrs old, perhaps corroborated by two samples collected at ∼4.4 m in Konggu, which yielded ages of ∼815 yrs (Fig. 3a, Supplementary Table 2). As the samples collected for analysis were selected based on their relative lack of secondary alteration, we estimate a maximum erosion-based age uncertainty of 25 yrs (smaller depending on the overall age of the sample). For the purpose of paleoseismological constraints, samples having ^230^Th ages that are outside the combined U-Th error and erosion-based uncertainty should be considered as documenting independent uplift events before the 2007 *M*_w_ 8.1 event.

The 2007 coseismic uplift profile along the long axis of Ranongga follows a fairly smooth classic elastic strain release curve because of slip on a reverse fault^1^,^31^. This smoothly varying uplift contrasts with the abruptly varying amounts of long-term net uplift quantified by maximum elevations of the mid-Holocene Ranongga coral terrace along the same line (Fig. 3a). Despite large amounts of 2007 coseismic uplift, the net Holocene emergence, and hence, the net uplift rate, are low at certain locations along the profile (Figs 1 and 3). For example, Lale, the site closest to the trench (labelled 1 in Fig. 1), experienced up to 2.4 m of coseismic uplift in 2007 but only has ∼7 m of net mid-Holocene emergence yielding uplift rates of 0.5–1.2 mm per yr (Fig. 3a). Here, coral microatolls uplifted during 2007 showed large amounts of pre-2007 submergence at rates on the order of ∼20 mm per yr (Fig. 3b), a result consistent with anecdotal evidence of dramatic coastal drowning during the past century. Despite an extensive search, we did not find any late Holocene *in situ* corals (0–5 ka) at the site. Thus, we infer that much of the uplift imposed by prehistoric earthquakes and that of the 2007 uplift at Lale is balanced by subsidence in the years preceding each megathrust earthquake as found at margins where there are large coseismic uplifts but very little net Holocene uplift^15^.

### Variable permanent uplift accumulation

In sharp contrast, north of Lale, it appears that large amounts of uplift do accumulate, leading to higher net uplift rates and the preservation of *in situ* corals documenting paleo-earthquakes. At locations such as Konggu, Newbare and Kolomali, we find a high mid-Holocene coral terrace (for example, 35 m at Konggu; 19 m at Kolomali; Fig. 3a) and coral microatolls that show very limited amounts of pre-2007 submergence (Fig. 3c). The *in situ* corals record the sum of permanent uplifts since the mid-Holocene. This indicates that the sum of interseismic subsidence and/or other tectonic processes that occur between great earthquakes may be vital to the geomorphic signature of uplift on forearcs. It is possible that much or most of the long-term uplifts occur steadily throughout the interseismic period and cancel much of the interseismic subsidence signal tied to elastic strain accumulation. At these locations farther from the trench than Lale, subsidence, the sum of both the elastic strain produced by interplate coupling and the long-term uplift accumulated over secular timescales, is insufficient to balance coseismic uplift. The net result is that a large component of uplift is retained, perhaps as the vertical component of general anelastic deformation within the upper plate.

The longest continuous sequence of coral levels from sea level inland and upward towards the mid-Holocene terrace summit is at Newbare, a location where uplift is retained. Here we used the difference between the heights of successive *in situ* coral levels to estimate the amount of abrupt uplift that has been retained as permanent uplift after each event. This exercise indicates that prehistoric earthquakes imposed uplifts that are comparable to or larger than the 2007 event (Fig. 4), with no consistent period of recurrence (Supplementary Table 5). It is possible that smaller abrupt events were not preserved in the coral record because of insufficient uplift, and subsequent exposure to excessive bioerosion (Fig. 3c). However, our methodology reliably captures large and abrupt events.

## Discussion

Taken together, the coral paleogeodesy data show that infrequent, but large abrupt uplift events have occurred in the past where the 1 April 2007 *M*_w_ 8.1 event involved interplate slip on the order of 5–10 m (refs 1, 2, 3, 4) and produced ≥2.5 m of uplift in parts of the western Solomons. Unless the 2007 event was unusual, by analogy, the large, older uplift events might have involved up to 10–15 m of horizontal slip on the plate interface. Using these estimates, the paleo-uplift record accounts for a maximum total of 90 m of coseismic slip over the last 2000 years (if we assume a maximum of six events with 15 m of slip per event), while the Australian plate converged by >190 m (on the basis of current convergence rates^9^). Thus, there are too few events in our coral record to accommodate the current Australian-Pacific convergence rate of ∼97 mm per yr (ref. 9). This implies that, apart from large coseismic megathrust rupture, additional mechanisms such as smaller ruptures, aseismic creep^20^, splay faulting^32^ and/or slow slip events^33^ must help to accommodate at least half of the interplate convergence.

Acceleration of late Quaternary uplift^11^, the irregular timing of large uplifts and, particularly, the variability of paleo-uplift geography show that the upper plate deformation response to subduction is not constant. This is in contrast to evidence from other islands near sediment-rich subduction zones such as in western Sumatra^17^ or Ramaree Island^16^ where the forearc persistently exhibits dominantly elastic behaviour and where net Holocene uplift rates are low. In the western Solomons, something must change from one event to the next because repeating slip on an identical fault should produce similar deformations of the upper plate. Considering the rapid convergence rates (up to 100 km per Myr) and rugged topography of the downgoing plate in the region, one potential explanation for our observations would be if the asperity patchiness changes from one slip event to the next^20^,^34^. Perhaps the asperities that were strong over one earthquake cycle are not the strongest ones over the next cycle^20^,^34^. If permanent upper plate strain occurs where locking is stronger and shear forces are concentrated then any change in asperities could show up in the deformation geography. It is also possible that slip can follow more than one fault plane and thus variable upper plate deformation simply reflects the differences in the distribution of forces and strain from one event to the next.

How can the western Solomons' case contribute to knowledge of the relationship between short-term seismicity and long-term deformation? Our observations of spatially and temporally varying permanent deformation and the importance of interseismic processes support the inference that the interplate thrust is likely to be complex rather than an idealized, gently curving surface^35^,^36^. When the upper plate is thin and heterogeneous in composition and strength, the patchiness of coupling during and between ruptures propagates to the surface as differential vertical deformation. Thus, it is important that this interplate thrust be visualized as a fault zone, and not simply a fault plane: every time the system accommodates slip, a new set of cards is dealt. Although such a situation may or may not be unique to this region, coral paleogeodesy can provide detailed histories of vertical deformation at locations accruing uplift that can provide a foundation for such hypotheses. These data can be used in conjunction with laboratory and modelling studies to test these hypotheses and investigate the mechanistic relationships between the accommodation of plate convergence, permanent deformation and seismicity^37^,^38^,^39^,^40^.

In summary, our results detail the complexity of the relationship between the short-term elastic earthquake cycle and longer-term permanent tectonic deformation above this shallow subduction interface. Although our data do not allow us to infer specific mechanisms, they strongly indicate that vertical motions of the land occurring between great earthquakes, either as interseismic elastic deformation, long-term background processes or, as the sum of both, play an essential role in the resulting topography of forearcs. Other forearcs could exhibit similar behaviour, but due to the greater depth from the surface to interplate thrust zones and the absence of land overlying most convergent margins, it is rare that well-dated paleoseismic uplift records can be developed so close to the trench. The results also illustrate the value of pre-instrumental records of land motions in forearcs. More geophysical campaigns in the western Solomons and other forearcs where coral-fringed islands overlie the megathrust would go far towards fully exploiting these uncommon windows into the shallow subduction interface.

## Methods

### U-Th analysis

Samples for U-Th dating were run on a Thermo-Finnigan ‘Neptune' multi-collector inductively coupled mass spectrometer located at the High-Precision Mass Spectrometry and Environment Change Laboratory, Department of Geosciences, National Taiwan University. Samples weigh between 0.05 and 0.16 g and were pretreated according to regular protocols (See Supplementary Table 1) prescribed in the literature^24^,^25^,^41^.

### Coral sample selection and uncertainty assessment

**Figure 2:**
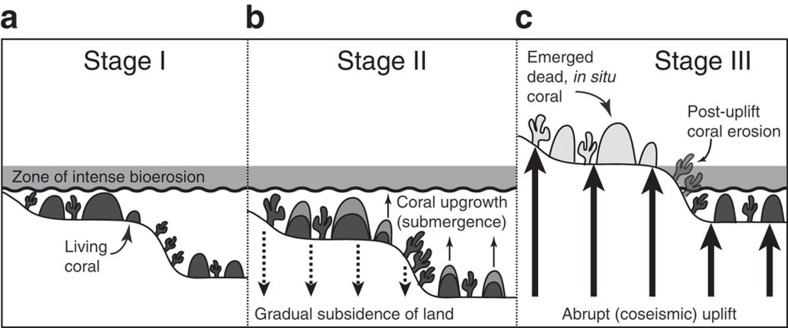
Illustration of the effect of vertical motions during an earthquake cycle on the coral record. (**a**) Stage 1: Living coral (dark grey) grows up until its highest level of survival (HLS) during a time period when the sea level is stable. (**b**) Stage II: In the years to decades preceding an earthquake, gradual interseismic subsidence submerges the live corals and allows them to grow vertically, as their HLS is raised. (**c**) Stage III: Significant, abrupt coseismic uplift raises the live reef above the HLS and kills the shallow-living corals. The corals lifted well above the new zone of intense bioerosion are preserved with intact morphology, whereas those just above the HLS undergo post-uplift erosion through bioerosion and eventually are not preserved.

**Figure 3:**
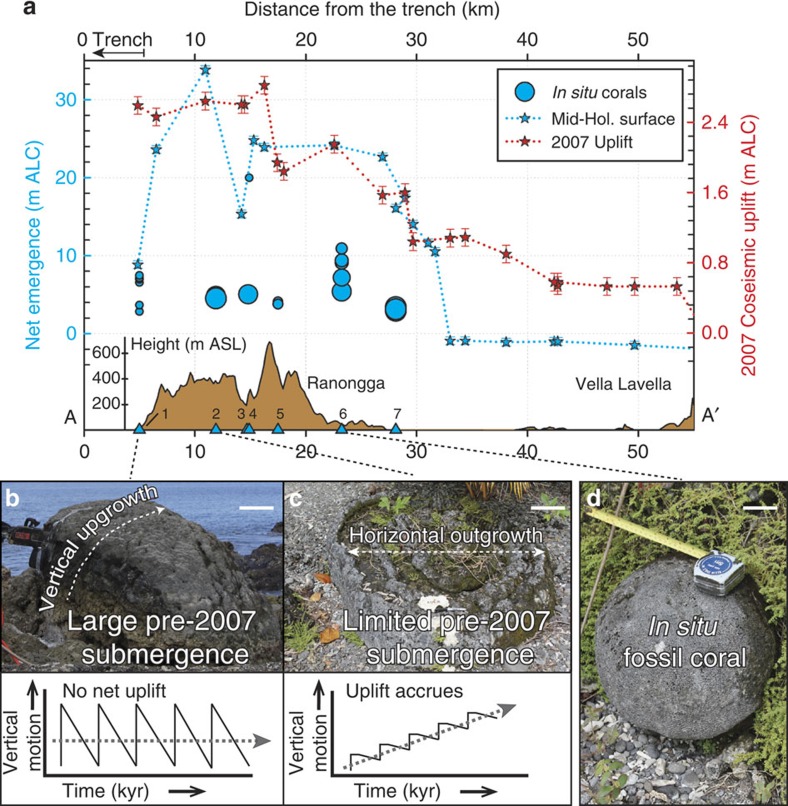
Coral-based uplift profile over three timescales on Ranongga and Vella Lavella islands. (**a**) Along path AA′ (Fig. 1), with trench to the left, comparison of net mid-Holocene emergence (blue stars; left axis), coseismic uplift pattern during the 2007 *M*_w_ 8.1 earthquake (red stars; right axis) and elevations of uplifted, *in situ* corals sampled (blue circles, sizes decreasing with age; left axis). Locations (blue triangles below) of sampling sites are plotted below along a profile of highest elevation on the islands along AA′; numbers indicate map locations labelled in Fig. 1. All elevation measurements are in metres above living coral (m ALC) except the topography, which is in metres above the mean sea level (ASL). Note: different sized blue circles (*in situ* coral samples) indicate different ages where sizes decrease with age. (**b**,**c**) Comparison of recently killed coral microatolls at Lale (**b**) and Konggu (**c**) that reveal vertical motion before the 2007 event along with an illustrative graph of the deformation profiles at their locations on a thousands-of-years timescale (below). The Lale microatoll indicates large pre-2007 submergence (that is, large interseismic subsidence before 2007 event), whereas the Konggu microatoll, ∼10 km north of Lale, shows a marked lack of pre-2007 submergence (that is, suppressed interseismic subsidence before the 2007 event). (**d**) A typical example of sampled *in situ* fossil coral with intact, well-preserved morphological characteristics at Konggu. Scale bars, (**b**) 25 cm, (**c**) 15 cm, and (**d**) 20 cm.

**Figure 4:**
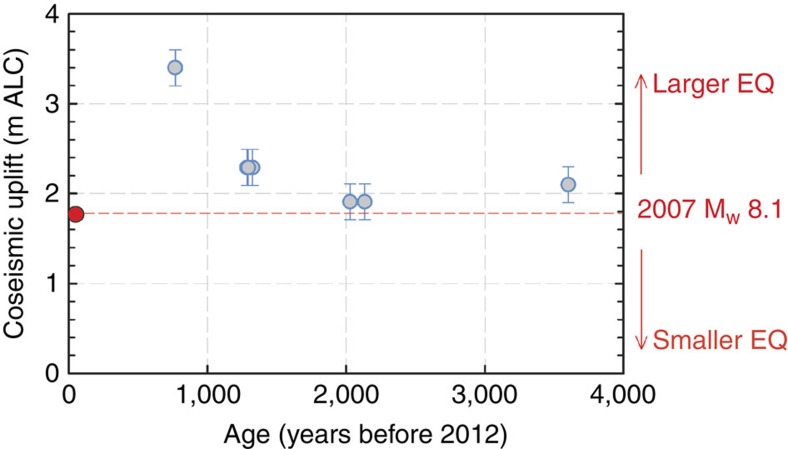
Coral paleoseismology at Newbare. Inferred amounts of coseismic uplift from the Newbare *in situ* coral record based on the relative elevation difference between subsequent vertical levels. Error bars are based on repeated bubble-level measurements (2*σ*=±0.5 m, *P*<0.05).

To ensure the paleo-uplift significance of our samples, we chose *in situ* corals that had retained their original, intricate morphology (for example, branches of *Acropora* sp. were intact; Supplementary Fig. 2a). We report the elevations of these *in situ* corals in metres above living coral (m ALC). By *in situ* we refer to corals found attached in growth position (Figs 2 and 3d, Supplementary Fig. 2). These corals were raised intact from their habitat below the sea level above the intertidal to supratidal zone of intense bioerosion (Fig. 2). Between these levels of *in situ* corals are parallel intervals barren of distinct corals. It is primarily the horizontal continuity parallel to the shoreline of *in situ* corals within a consistent vertical interval, rather than terrace-like morphology, by which we recognized the series of coral levels. The shoreline probably remained too briefly at any given level for morphologic terraces to form except for the well-defined uppermost mid-Holocene terrace surface. We sampled *in situ* corals on the eastern coast of Ranongga from 1.5 to 20 m elevation along the seaward slope from the sea level towards the higher Holocene terrace flat (Fig. 1). Before uplift, the zone above each level of corals was subjected to intense bioerosion from about the mean sea level upwards to 0.5 m above the mean high water depending on wave energy at any specific site. This would account for the smoothed surfaces devoid of freestanding corals just above each level of *in situ* coral (Fig. 2).

A sequence of small uplifts rather than a single large uplift could produce a coral level at a particular elevation. However, if the next uplift did not occur within a few years, a coral uplifted less than 1 m would be subject to intense bioerosion and would soon be destroyed, unless protected by some extraordinary conditions such as burial beneath sand (see Stage III in Fig. 2). Although minor surface erosion after coral death increases the overall age uncertainty above the analytical error of the U-Th disequilibrium ages, the intact coral morphology dictates that these errors are minor (<20–40 years including analytical uncertainty dependent on the age of the coral) and insignificant to our interpretation. Although aseismic uplift is possible in this setting, and could add to total uplift, it would have to exceed 1.5 m within a span of a few years for the coral morphology to survive its transit to safety above high tide level and bioerosion.

Despite the southwest Pacific regional sea level having fallen by ∼2 m because of ‘ocean siphoning' since ∼6 ka (ref. 42) there is no evidence that it ever fell abruptly at a fast enough rate that would not only kill but allow delicate corals to escape marine erosion as they emerged. Replication of the U-Th ages of independent, *in situ* coral samples from the same vertical level bolsters the robustness of our interpretation and the reconstructed uplift profile on Ranongga Island (Supplementary Tables 1 and 2).

## Additional information

**How to cite this article:** Thirumalai, K. *et al.* Variable Holocene deformation above a shallow subduction zone extremely close to the trench. *Nat. Commun.* 6:7607 doi: 10.1038/ncomms8607 (2015).

## Supplementary Material

Supplementary Information Supplementary Figures 1-2, Supplementary Tables 1-5 and Supplementary References
