# Supplementary material for: Variable Holocene deformation above a shallow subduction zone extremely close to the trench
Source: Nat Commun. 2015 Jun 30;6:7607. doi: 10.1038/ncomms8607 (PMC4491809; doi:10.1038/ncomms8607)
Supplement: Supplementary Information — Supplementary Figures 1-2, Supplementary Tables 1-5 and Supplementary References [file ncomms8607-s1.pdf]

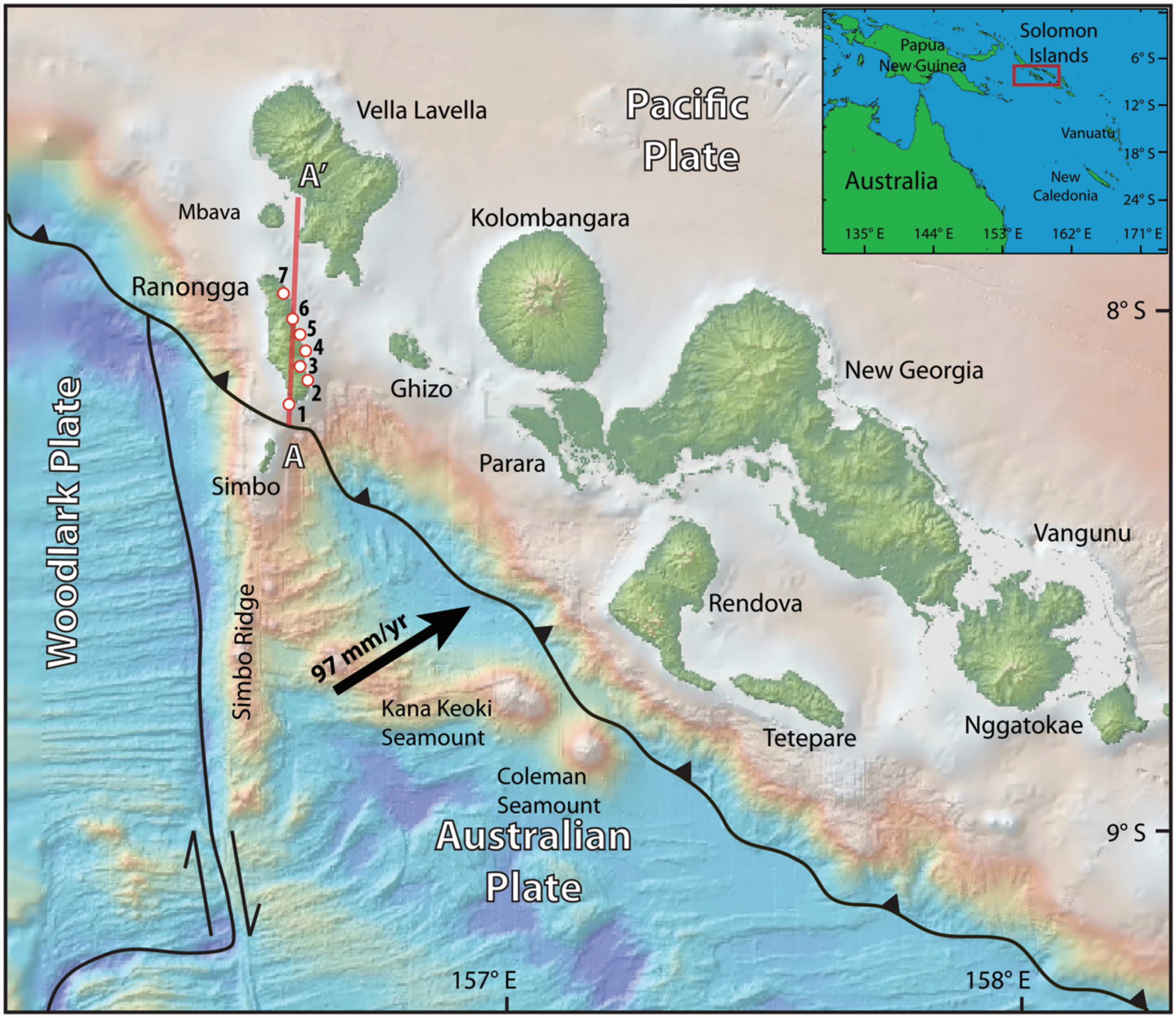

Supplementary Fig. 1. Larger map of tectonic setting (and bathymetry) of the western Solomon subduction interface. Sampling sites on Ranongga are depicted by white circles where 1 - Lale, 2 - Konggu, 3 - Ndae, 4 - Perava Pt., 5 - Ena, 6 - Newbare, 7 - Kolomali. AA′ depicts the transect used in Fig. 1. The Australian plate containing rigid asperities in the Kana Keoki and Coleman Seamounts subducts beneath the Pacific Plate. The bathymetric base map is from Supplementary Ref. 1.

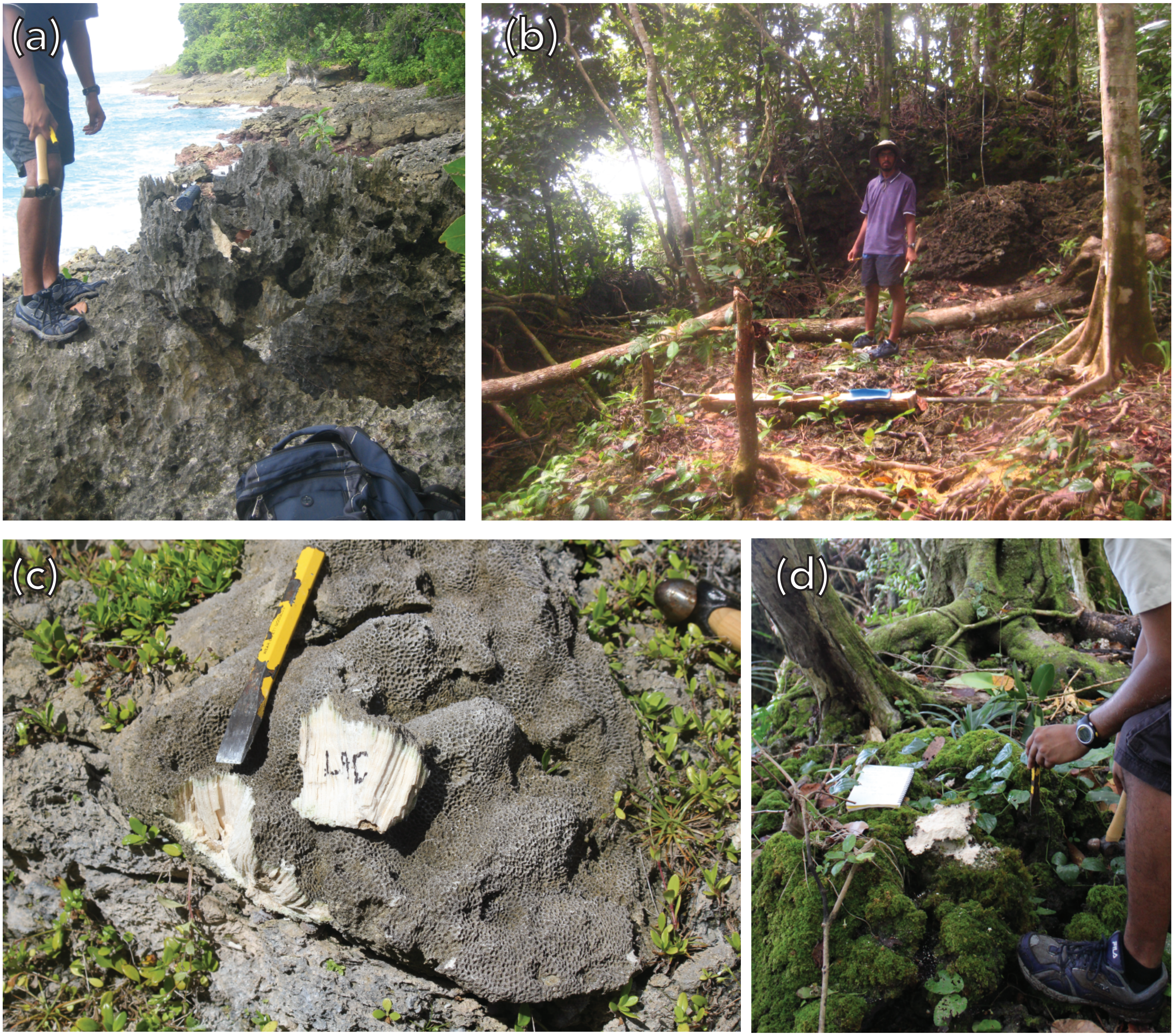

Supplementary Fig. 2. Field photographs from Ranongga Island: (a) Uplifted, *in-situ Acropora palmata* samples at Ndae (3 from Fig. 1) (b) A vertical level at Newbare (6 in Fig. 1; 7.68 m ALC) where we collected samples 12-NB-C, 12-NB-D, and 12-NB-E that dated to ~1290 yrs ago. (c) Uplifted *in-situ* coral at Lale (1 in Fig. 1; sample 12-LA-C) that was dated to be ~6,000 yrs in age, corroborating other coral ages proximal to this sample. Note the minimal amount of erosion. (d) Moss-covered but internally pristine coral samples at Perava Point (4 in Fig. 1) that yielded mid-Holocene ages.

Photography Credits:
(a) Fred Taylor (b) Fred Taylor (c) Kaustubh Thirumalai (d) Alison Papabatu

Supplementary Table 1. Sample Ages. Uranium and thorium isotopic compositions and ^{230}Th ages[#] for *in-situ* coral samples ordered by location number in Figure 1 of the main text.

Sample ID	Mass (g)	^{238}U (ppb)	^{232}Th (ppt)	δ^{234}U measured[a]	[^{230}Th/^{238}U] activity[c]	[^{230}Th/^{232}Th] (ppm[d])	Age uncorrected	Age corrected[c,e]	$\delta^{234}U_{initial}$ corrected[b]
1. 12-LA-B	0.1154	2354.6 ± 2.2	626.6 ± 6.1	140.6 ± 1.5	0.0668 ± 0.00011	4147 ± 41	6,587 ± 14	6,581 ± 15	143.2 ± 1.5
1. 12-LA-C	0.0961	2400.5 ± 2.3	1270.3 ± 7.8	141.5 ± 1.5	0.0608 ± 0.00015	1898 ± 12	5,973 ± 17	5,960 ± 18	143.9 ± 1.5
1. 12-LA-D	0.0769	2309.5 ± 2.0	55.4 ± 9.1	141.5 ± 1.4	0.0641 ± 0.00015	44061 ± 7198	6,298 ± 18	6,298 ± 18	144.0 ± 1.4
1. 12-LA-E	0.0894	2757.6 ± 1.8	252.9 ± 7.8	143.9 ± 1.3	0.0629 ± 0.000084	11323 ± 349	6,169 ± 11	6,167 ± 11	146.5 ± 1.3
1. 12-LA-F	0.0662	2381.2 ± 2.3	222 ± 11	143.3 ± 1.3	0.0661 ± 0.00011	11689 ± 554	6,490 ± 13	6,488 ± 13	145.9 ± 1.3
1. 12-LA-G	0.0733	2255.5 ± 2.6	152.2 ± 9.5	143.5 ± 1.7	0.0636 ± 0.00015	15562 ± 972	6,240 ± 18	6,239 ± 18	146.1 ± 1.7
2. 12-KON-A	0.0740	2392.8 ± 2.3	236.5 ± 9.4	145.3 ± 1.5	0.0003 ± 0.000010	44.5 ± 2.4	25.42 ± 0.93	23.1 ± 1.5	145.3 ± 1.5
2. 12-KON-B	0.1598	1975.7 ± 2.8	5.9 ± 4.4	143.0 ± 2.1	0.0003 ± 0.0000091	1566 ± 1158	27.03 ± 0.87	26.96 ± 0.87	143.0 ± 2.1
2. 12-KON-C	0.0646	2622.1 ± 1.7	240.0 ± 10.8	149.1 ± 1.4	0.0002 ± 0.000011	29 ± 2	15.4 ± 1.0	13.3 ± 1.5	149.1 ± 1.4
2. 12-KON-C	0.0845	2590.6 ± 3.9	120.2 ± 8.2	146.2 ± 2.1	0.0002 ± 0.0000093	55.0 ± 5.0	14.72 ± 0.89	13.7 ± 1.0	146.2 ± 2.1
2. 12-KON-D	0.0973	2674.3 ± 2.0	90.7 ± 7.2	149.9 ± 1.4	0.0086 ± 0.000034	4202 ± 332	823.0 ± 3.4	822.2 ± 3.4	150.2 ± 1.4
2. 12-KON-E	0.0574	2501.8 ± 2.8	1552 ± 13	142.6 ± 1.5	0.0086 ± 0.000048	229.3 ± 2.2	826.5 ± 4.7	812.1 ± 8.6	142.9 ± 1.5
2. 12-KON-F	0.1056	2694.5 ± 4.0	44.5 ± 4.4	145.1 ± 1.6	0.0064 ± 0.000027	6425 ± 635	614.6 ± 2.7	614.3 ± 2.7	145.4 ± 1.6
3. 12-PER-A	0.1773	1923.0 ± 2.7	12.3 ± 2.6	142.6 ± 1.8	0.0749 ± 0.000151	193375 ± 41214	7,384 ± 20.0	7,384 ± 20.0	145.6 ± 1.8
4. 12-ENA-A	0.0613	2663.3 ± 3.4	327 ± 11	145.8 ± 1.8	0.0091 ± 0.000044	1226 ± 43	872.0 ± 4.4	869.2 ± 4.6	146.2 ± 1.8
5. 12-DAE-A	0.0707	2108.4 ± 2.3	29 ± 10	142.0 ± 1.4	0.0401 ± 0.00034	47658 ± 16031	3,900 ± 34	3,899 ± 34	143.6 ± 1.4
5. 12-DAE-B	0.0789	2206.1 ± 3.5	149.7 ± 8.8	143.7 ± 2.2	0.0401 ± 0.00011	9743 ± 575	3,889 ± 13	3,888 ± 13	145.3 ± 2.2
6. 12-NB-B	0.1223	2447.4 ± 2.9	11.6 ± 5.7	146.5 ± 1.7	0.0080 ± 0.000032	28112 ± 13840	768.6 ± 3.2	768.5 ± 3.2	146.8 ± 1.7
6. 12-NB-C	0.0806	2181.8 ± 3.0	82.4 ± 8.6	146.7 ± 1.8	0.0138 ± 0.000053	6044 ± 634	1,324.9 ± 5.6	1,324.1 ± 5.6	147.3 ± 1.8
6. 12-NB-D	0.1263	2520.6 ± 2.1	42.4 ± 5.5	142.3 ± 1.5	0.0134 ± 0.00011	13128 ± 1711	1,285 ± 11	1,285 ± 11	142.8 ± 1.5
6. 12-NB-E	0.0922	2239.6 ± 2.9	19.4 ± 7.6	146.3 ± 1.8	0.0135 ± 0.000050	25733 ± 10001	1,295.2 ± 5.3	1,295.0 ± 5.3	146.8 ± 1.8
6. 12-NB-F	0.1265	2322.5 ± 3.1	79.6 ± 5.5	146.1 ± 1.9	0.0080 ± 0.000036	3861 ± 267	766.5 ± 3.7	765.7 ± 3.7	146.5 ± 1.9

6. 12-NB-G	0.1338	2396.3 ± 2.5	23.3 ± 5.2	145.0 ± 1.7	0.0211 ± 0.000070	35752 ± 7976	2,028.2 ± 7.4	2,028.0 ± 7.4	145.8 ± 1.7
6. 12-NB-H	0.0875	2085.5 ± 2.4	38.9 ± 8.0	142.7 ± 1.7	0.0221 ± 0.000065	19597 ± 4012	2,133.7 ± 7.2	2,133.3 ± 7.2	143.6 ± 1.7
6. 12-NB-K	0.1172	2712.8 ± 2.3	444.5 ± 6.0	147.6 ± 1.2	0.0373 ± 0.000176	3757 ± 54	3,605.0 ± 17.9	3,601.2 ± 17.9	149.1 ± 1.3
7. 13K-E	0.10550	2126.2 ± 2.0	226.8 ± 4.4	143.8 ± 1.5	0.00887 ± 0.000025	1371 ± 27	849.0 ± 2.7	846.6 ± 3.0	144.1 ± 1.5
7. 91RAN-G1a	0.1387	2123.8 ± 1.8	4.9 ± 5.0	147.7 ± 1.7	0.0061 ± 0.000025	43662 ± 44430	585.8 ± 2.5	585.7 ± 2.5	148.0 ± 1.7
7. 91RAN-G3	0.1133	2169.3 ± 1.5	26.5 ± 6.1	146.2 ± 1.3	0.0063 ± 0.000024	8558 ± 1988	603.7 ± 2.4	603.5 ± 2.4	146.5 ± 1.3

[#] Chipped subsamples were cleaned[2] for U-Th chemistry[3] and isotopic measurements on a multi-collector inductively coupled plasma mass spectrometer (MC-ICP-MS), Thermo Electron Neptune, at HISPEC, NTU[4]. A gravimetrically calibrated triple-spike, ^{229}Th-^{233}U-^{236}U, isotope dilution method was employed to correct mass bias and determine U-Th contents and isotopic compositions[5]. Analytical errors are 2σ of the mean.

[a] $\delta^{234}U = ([^{234}U/^{238}U]_{activity} - 1) \times 1000$.

[b] $\delta^{234}U_{initial}$ corrected was calculated based on the ^{230}Th age (T), i.e., $\delta^{234}U_{initial} = \delta^{234}U_{measured}\ X\ e^{\lambda_{234}{}^{*}T}$; where T is the corrected age and λ is the decay constant.

[c] $[^{230}Th/^{238}U]_{activity} = 1 - e^{-\lambda_{230}T} + (\delta^{234}U_{measured}/1000)[\lambda_{230}/(\lambda_{230} - \lambda_{234})](1 - e^{-(\lambda_{230} - \lambda_{234})T})$, where T is the age; Decay constants are 9.1577×10^{-6} yr^{-1} for ^{230}Th, 2.8263×10^{-6} yr^{-1} for ^{234}U (Supplementary Ref. 5), and 1.55125×10^{-10} yr^{-1} for ^{238}U (Supplementary Ref. 6).

[d] The degree of detrital ^{230}Th contamination is indicated by the $[^{230}Th/^{232}Th]$ atomic ratio instead of the activity ratio.

[e] Age (before the chemistry date of May 2013) corrections were calculated using an estimated atomic $^{230}Th/^{232}Th$ ratio of 4 (± 2) ppm (Supplementary Ref. 8). There is no significant age difference between the determined ages and ones calculated with the recent new ^{230}Th and ^{234}U decay constants[7].

Supplementary Table 2. Paleogeodetic Observations and Results. Net emergence is calculated as the difference between the elevation and relative paleosea level[8,9]. Samples are ordered based on numbering in Fig. 1. At a particular location, colored highlighting is used to indicate samples from the same vertical levels. All coral samples can be found within ± 10 m of these coordinates and within ± 0.5 m of the reported elevations. Uncertainty in relative paleosea level and dating errors are negligible to our interpretation. *

Cautionary Note: Colors are indicative of same vertical level at that location ONLY (not in between locations)

Sl.No.	Sample	Location	GPS Coordinates	Elevation (m ALC)#	Age (yrs prior to 2012)	± 2σ Error (yrs)	Relative Paleosea Level (m)	Net Emergence (m ALC)#
1	12-LA-C	1. Lale	(8.182°S,156.579°E)	4.84	5960	± 18.0	+2	2.84
2	12-LA-B	1. Lale	(8.182°S,156.579°E)	5.66	6581	± 15.0	+2	3.66
3	12-LA-D	1. Lale	(8.180°S,156.579°E)	8.56	6298	± 18.0	+2	6.56
4	12-LA-E	1. Lale	(8.180°S,156.579°E)	8.96	6167	± 11.0	+2	6.96
5	12-LA-F	1. Lale	(8.180°S,156.579°E)	9.16	6488	± 13.0	+2	7.16
6	12-LA-G	1. Lale	(8.180°S,156.579°E)	9.46	6239	± 18.0	+2	7.46
7	12-KON-A	2. Konggu	(8.128°S,156.613°E)	2.54	23.1	± 1.5	0	3.04
8	12-KON-B	2. Konggu	(8.128°S,156.613°E)	2.61	26.96	± 0.9	0	2.99
9	12-KON-C	2. Konggu	(8.128°S,156.613°E)	2.58	13.7	± 1.0	0	3.26
10	12-KON-F	2. Konggu	(8.128°S,156.613°E)	4.17	614.3	± 2.7	0	4.52
11	12-KON-D	2. Konggu	(8.128°S,156.613°E)	4.44	822.2	± 3.4	0	4.79
12	12-KON-E	2. Konggu	(8.128°S,156.613°E)	4.49	812.1	± 8.6	0	4.84
13	12-PER-A	3. Perava	(8.099°S,156.608°E)	16.99	7384	± 20.0	-3	19.99
14	12-ENA-A	4. Ena	(8.096°S,156.606°E)	5.04	869.2	± 4.6	0	5.04
15	12-DAE-D	5. Ndae	(8.070°S,156.603°E)	2.1	10.2	± 1.2	0	2.1
16	12-DAE-A	5. Ndae	(8.070°S,156.603°E)	5.6	3899	± 34.0	+1.5	4.1
17	12-DAE-B	5. Ndae	(8.070°S,156.603°E)	5.6	3888	± 13.0	+1.5	4.1
18	12-DAE-C	5. Ndae	(8.070°S,156.603°E)	5.3	3831	± 11.0	+1.5	3.8
19	12-NB-B	6. Newbare	(8.012°S,156.580°E)	5.39	768.5	± 3.2	0	5.39
20	12-NB-F	6. Newbare	(8.012°S,156.580°E)	5.39	765.7	± 3.7	0	5.39
21	12-NB-C	6. Newbare	(8.012°S,156.580°E)	7.68	1324.1	± 5.6	+0.5	7.18
22	12-NB-D	6. Newbare	(8.012°S,156.580°E)	7.68	1285	± 11.0	+0.5	7.18
23	12-NB-E	6. Newbare	(8.012°S,156.580°E)	7.68	1295	± 5.3	+0.5	7.18
24	12-NB-G	6. Newbare	(8.012°S,156.580°E)	9.59	2028	± 7.4	+0.5	9.09
25	12-NB-H	6. Newbare	(8.012°S,156.580°E)	9.89	2133.3	± 7.2	+0.5	9.39
26	12-NB-K	6. Newbare	(8.012°S,156.580°E)	11.99	3601	± 18.0	+1	10.99
27	RAN-G-3	7. Kolomali	(7.961°S,156.603°E)	2.93	603.5	± 2.4	0	2.93
28	RAN-G-1a	7. Kolomali	(7.961°S,156.603°E)	3.13	585.7	± 2.5	0	3.13
29	13K-E	7. Kolomali	(7.961°S,156.603°E)	3.40	846.6	± 3.0	0	3.43

# - Error on elevation is ± 0.5 m (2σ) based on repeated bubble-level measurements

* - The sea level regressions concerning our study are of the order of 2 m or more. No such abrupt sea level falls exist that could leave behind preserved corals in the time period of interest. The island size effect of Ranongga, Vella Lavella and other smaller islands is negligible[10]. El Niño Southern Oscillation events and modern sea-level rise produce variability on the order of ≤0.5 m (Supplementary Ref. 11) and could contribute 0.2-0.3 m toward the coral record and might explain some of the intra-level age variability, but are insignificant to our interpretation and negligible to inter-level age and elevation differences.

Supplementary Table 3. Amount of Coseismic Uplift during 2007 Event on Ranongga and Vella Lavella Islands (Supplementary Ref. 12 and this study).

Location		Coseismic Uplift in 2007 (m)
Latitude	Longitude	
-7.726	156.528	0
-7.749	156.553	0.53
-7.783	156.558	0.53
-7.805	156.520	0.53
-7.845	156.534	0.58
-7.845	156.534	0.54
-7.848	156.546	0.58
-7.886	156.499	0.90
-7.919	156.526	1.09
-7.931	156.526	1.08
-7.961	156.565	1.04
-7.968	156.568	1.60
-7.986	156.577	1.57
-8.025	156.583	2.15
-8.065	156.601	1.84
-8.071	156.603	1.94
-8.081	156.604	2.82
-8.097	156.607	2.6
-8.099	156.608	2.6
-8.128	156.613	2.64
-8.168	156.603	2.46
-8.183	156.579	2.59

Supplementary Table 4. Mid-Holocene Emergence (Supplementary Ref. 13 and this study). Relative paleosea level of +2 m was used to obtain net emergence from elevations.

Location		Net Emergence (m)
Latitude	Longitude	
-7.726	156.528	0
-7.783	156.558	0.53
-7.845	156.534	1
-7.845	156.534	1
-7.848	156.546	1
-7.886	156.499	0.9
-7.919	156.526	1.09
-7.931	156.526	1.08
-7.943	156.547	12.54
-7.949	156.559	13.64
-7.961	156.565	16.04
-7.975	156.572	18.1
-7.968	156.568	19.4
-7.986	156.577	24.67
-8.025	156.581	26.15
-8.081	156.604	25.92
-8.089	156.602	26.8
-8.099	156.608	17.35
-8.128	156.613	35.84
-8.168	156.603	25.6
-8.183	156.579	10.84

Supplementary Table 5. Newbare Paleoseismology. Residual uplift was calculated based on the difference in heights between subsequent levels. Residuals for the first level were calculated with the 2007 uplift at Newbare (~1.99 m).

Sl. No.	Sample Name	Elevation (m ALC)	U-Th Age (years)	Residual Uplift (m ALC)
1	12-NB-B	5.39	768.5	3.40
2	12-NB-F	5.39	765.7	3.40
3	12-NB-C	7.68	1324.1	2.29
4	12-NB-D	7.68	1285	2.29
5	12-NB-E	7.68	1295	2.29
6	12-NB-G	9.59	2028	1.91
7	12-NB-H	9.89	2133.3	1.91
8	12-NB-K	11.99	3601	2.10

Supplementary References

1. Ryan, W. B. F. *et al.* Global Multi-Resolution Topography synthesis. *Geochem. Geophys. Geosyst.* **10,** 1–9 (2009)
2. Shen, C.-C. *et al.* Variation of initial $^{230}Th/^{232}Th$ and limits of high precision U–Th dating of shallow-water corals. *Geochim. Cosmochim. Ac.* **72,** 4201–4223 (2008).
3. Shen, C.-C. *et al.* Measurement of attogram quantities of ^{231}Pa in dissolved and particulate fractions of seawater by isotope dilution thermal ionization mass spectroscopy, *Anal. Chem.* **75**, 1075-1079 (2003).
4. Shen, C.-C. *et al.* High-precision and high-resolution carbonate ^{230}Th dating by MC-ICP-MS with SEM protocols. *Geochim. Cosmochim. Ac.* **99,** 71–86 (2012).
5. Cheng, H. *et al.* Improvements in ^{230}Th dating, ^{230}Th and ^{234}U half-life values, and U–Th isotopic measurements by multi-collector inductively coupled plasma mass spectrometry. *Earth Planet. Sc. Lett.* **371-372,** 82–91 (2013).
6. Jaffey, A. H., Flynn, K. F., Le, G., Bentley, W. C. & Essling, A. M. Precision Measurement of Half-Lives and Specific Activities of U-235 and U-238. *Phys. Rev. C* **4,** 1889–1905 (1971).
7. Cheng, H. *et al.* The half-lives of uranium-234 and thorium-230. *Chem. Geol.* **169,** 17–33 (2000).
8. Fleming, K. *et al.* Refining the eustatic sea-level curve since the Last Glacial Maximum using far-and intermediate-field sites. *Earth Planet. Sc. Lett.* **163,** 327–342 (1998).
9. Cabioch, G., Montaggioni, L. F., Faure, G. & Ribaud-Laurenti, A. Reef coralgal assemblages as recorders of paleobathymetry and sea level changes in the Indo-Pacific province. *Quat. Sci. Rev.* **18,** 1681–1695 (1999).
10. Mitrovica, J. X. & Milne, G. A. On the origin of late Holocene sea-level highstands within equatorial ocean basins. *Quat. Sci. Rev.* **21,** 2179–2190 (2002).
11. Merrifield, M. A., Thompson, P. R. & Lander, M. A. Multidecadal sea level anomalies and trends in the western tropical Pacific. *Geophys. Res. Lett.* **39,** L13602 (2012).
12. Taylor, F. W. *et al.* Rupture across arc segment and plate boundaries in the 1 April 2007 Solomons earthquake. *Nat. Geosci.* **1,** 253–257 (2008).
13. Mann, P., Taylor, F. W., Lagoe, M. B., Quarles, A. & Burr, G. S. Accelerating late Quaternary uplift of the New Georgia Island Group (Solomon island arc) in response to subduction of the recently active

Woodlark spreading center and Coleman seamount. *Tectonophysics* **295,** 259–306 (1998).
